# Computed tomographic quantitative evaluation of common bile duct size in normal dogs: A reference range study considering body weight

**DOI:** 10.3389/fvets.2023.1137400

**Published:** 2023-03-30

**Authors:** Yein Kim, Sung-Soo Kim, Danbee Kwon, Deokho Im, Kichang Lee, Hakyoung Yoon

**Affiliations:** ^1^Department of Veterinary Medical Imaging, College of Veterinary Medicine, Jeonbuk National University, Iksan, Republic of Korea; ^2^Department of Veterinary Medical Imaging, VIP Animal Medical Centre, Seoul, Republic of Korea; ^3^Department of Veterinary Medical Imaging, Bundang Leaders Animal Medical Centre, Seongnam, Republic of Korea; ^4^Department of Veterinary Medical Imaging, Nel Animal Medical Center, Anyang-si, Gyeonggi-do, Republic of Korea

**Keywords:** canine, computed tomography, biliary system, CBD diameter to Ao ratio, CBD size

## Abstract

**Introduction:**

Common bile duct (CBD) measurements are important for the evaluation of biliary systemic disorders. However, in veterinary medicine, reference ranges for specific body weights (BW) and correlation between CBD diameter and BW have not been studied. This study aimed to establish normal reference ranges of CBD diameter for different BW groups and to analyse correlation between CBD diameter and BW in dogs without hepatobiliary disease. Additionally, normal reference ranges of CBD to aorta ratio (CBD: Ao ratio) were established which is not affected by BW.

**Methods:**

CBD diameter was measured at three different sites: porta hepatis (PH), duodenal papilla (DP) level and mid-portion (Mid) between these points using computed tomography (CT) in 283 dogs without hepatobiliary disease.

**Results:**

The reference range of CBD diameter at PH level: 1.69 ± 0.29 mm (Class 1; 1 kg ≤ BW < 5 kg), 1.92 ± 0.35 mm (Class 2; 5 kg ≤ BW < 10 kg), 2.20 ± 0.43 mm (Class 3; 10 kg ≤ BW < 15 kg), 2.79 ± 0.49 mm (Class 4; 15 kg ≤ BW < 30 kg); Mid-level: 2.06 ± 0.25 mm (Class 1), 2.43 ± 0.37 mm (Class 2), 2.74 ± 0.52 mm (Class 3), 3.14 ± 0.44 mm (Class 4); DP level: 2.33 ± 0.34 mm (Class 1), 2.90 ± 0.36 mm (Class 2), 3.35 ± 0.49 mm (Class 3), and 3.83 ± 0.50 mm (Class 4). There was a significant difference in CBD diameter at each level among all BW groups. Furthermore, BW and CBD diameter showed positive linear correlation at each level. We devised CBD: Ao ratio at each level that showed no significant difference between the different BW groups; PH level: 0.34 ± 0.05; Mid-level: 0.42 ± 0.06; DP level: 0.47 ± 0.06.

**Conclusion:**

In conclusion, since the CBD diameter for each BW is significantly different, different normal reference ranges of CBD diameter should be applied for each BW, and the CBD: Ao ratio can be used regardless of the BW.

## 1. Introduction

Extrahepatic bile duct measurement is necessary to evaluate patients affected by cholestasis since this condition is associated with clinical complications such as injury of the liver and gallbladder (GB) and disruption of hepatic circulation (1–6). Common bile duct (CBD) dilation can be caused by obstructive or non-obstructive causes, but most are associated with obstructive causes (7). Extrahepatic biliary tract obstruction (EHBO) has been attributed to a variety of causes including pancreatitis, GB mucocele, pancreatobiliary malignancy, extrinsic luminal compression, obstruction due to intraluminal components (bile duct stones, foreign body, etc.), obstruction and stenosis of the duodenal papilla (DP), and fibrosing cholangitis (6, 8–10). In particular, chronic pancreatitis and cholangiohepatitis with choledocholithiasis are the most common causes of partial or complete EHBO (1, 8, 9).

In veterinary medicine, there are several studies that evaluate bile duct diameter in dogs (10, 11). One previous study evaluated CBD diameter using ultrasound (US) and the upper limit of the normal CBD diameter was defined as 3 mm (10). US has the advantage of being faster and cheaper and can be performed without anesthesia when evaluating the biliary tract. Therefore, US is often used as the first diagnostic tool for evaluation of the biliary system and frequent follow-up monitoring is also possible (8). However, US examination in small animals is more difficult than in humans because the bile duct is often within the rib cage and obscured by gas in the stomach or intestine (8). Therefore, visualization of the CBD may not always be possible and may be inconsistent. In addition, US may not be able to evaluate the entire CBD and the specificity and sensitivity can be greatly affected by the skill and experience of the sonographer (12). In contrast, evaluation of the extrahepatic bile duct using computed tomography (CT) can better visualize the anatomy and location of the extrahepatic bile duct than ultrasound (6, 13). Moreover, CT can be very useful in evaluating periductal mass lesions and the entire CBD length and diameter, in which evaluation of the entire CBD is important to diagnose partial obstruction (8, 13). Although there was a study on the CBD diameter of normal dogs using CT in veterinary medicine (11), to the best of the author's knowledge, there has been no study suggesting reference ranges for specific body weight (BW) groups and a correlation between BW, sex, age and CBD diameter in dogs. Also, weight-independent parameters have not been not identified.

Therefore, the aims of this study were as follows: (1) Establish a reference range for CBD diameter in dogs without hepatobiliary disease and identify the differences of CBD diameter between different BW groups; (2) identify the correlation between BW and CBD diameter; (3) evaluate CBD diameter to abdominal aorta (Ao) diameter ratio (CBD: Ao ratio) as a weight-independent parameter; and (4) analyse the significance of differences in CBD diameter in relation to age and sex.

## 2. Materials and methods

### 2.1. Animals

In this multicenter, retrospective, observational study, medical charts including complete blood counts, serum biochemistry, and CT images of 635 dogs were collected from four veterinary clinics (Jeonbuk National University Animal Medical Center, Bundang Leaders Animal Medical Center, VIP Animal Medical Center, and Nel Animal Medical Center) between 2 August 2021 and 20 December 2022. The inclusion criteria were normal serum chemistry results [white blood cell count, alkaline phosphatase (ALP) level, alanine aminotransferase (ALT) level, gamma glutamyl transferase (GGT) level, total bilirubin concentration, albumin concentration, and cholesterol level], no abnormalities on CT images (liver, biliary system, and pancreas), and no clinical signs related to liver and pancreatic disease (anorexia, vomiting, diarrhea, and abdominal pain). Dogs weighing over 30 kg were excluded from this study. In total, 283 dogs were included in the analysis and were classified into four groups based on their BW (Class 1: 1 kg ≤ BW < 5 kg, Class 2: 5 kg ≤ BW < 10 kg, Class 3: 10 kg ≤ BW < 15 kg, Class 4: 15 kg ≤ BW < 30 kg).

This study was approved by the Institutional Animal Care and Use committee of Jeonbuk National University (Approval No. JBNU 2022-037).

### 2.2. Measurement methods

CT images were acquired using three scanners (Revolution ACT or Brivo CT 385; GE Hangwei Medical System Co., Ltd., Beijing, China/Alexion 16; Toshiba Medical Systems Co Ltd., Otowara, Japan) with the following scan parameters: 120 kVp, 80–200 mAs, 0.625–1.25-mm slice thickness, 0.75–1.0-s rotation time, and a 0.938–1.375 collimation beam pitch. The scan field included at least the cranial margin of the diaphragm to the caudal endplate of the third lumbar vertebra, and scans were performed under breath-holding to minimize artifacts caused by movement due to breathing. All images were reviewed using an abdominal window (window level = 40 Hounsfield units [HU], window width = 400 HU) and a Radiant Dicom viewer (Medixant; Poznan, Poland). Portal venous and delayed phase images were used in all measurements for the best visualization of the liver, CBD, and other surrounding organs. Iohexol (Omnipaque, 750 mgI/kg; GE Healthcare, Ireland) was used for contrast medium and administered *via* cephalic vein.

**Figure 1:**
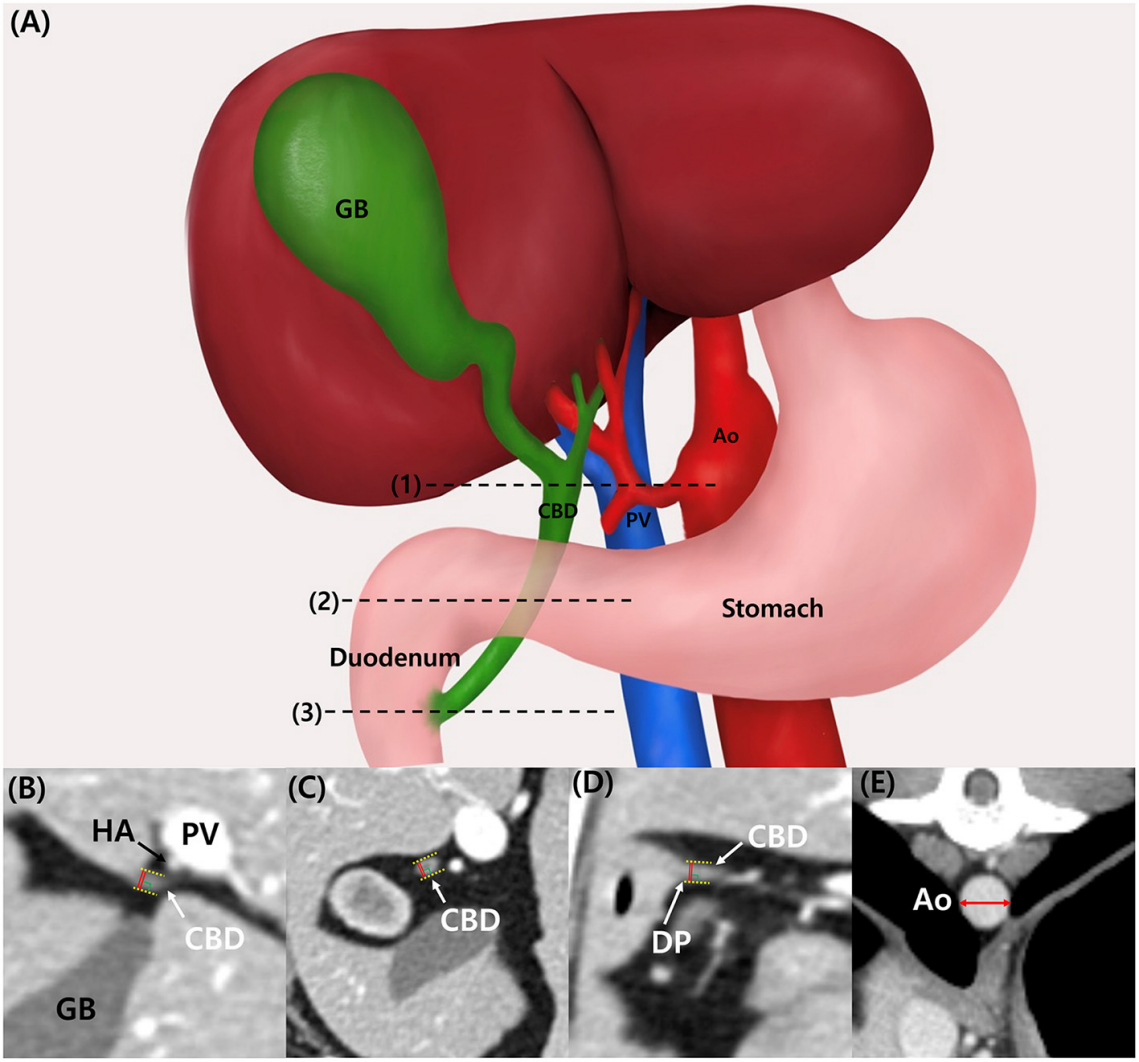
Illustration of common bile duct in dogs. **(A)** The location of measurement of common bile duct diameter (CBD) were as following: porta hepatis (PH) level (1), duodenal papilla (DP) level (3), and mid-portion (Mid) between these points (2). CT images of CBD at PH level **(B)**, Mid-level **(C)**, and DP level **(D)**. The measurement of CBD is made including the ductal wall (yellow dotted line), and the widest diameter was measured (red line) perpendicular to its long axis. CT images of aortic diameter (Ao) measurement **(E)**. For the Ao measurement, minor axis diameter (red double arrow) was measured. Ao, aorta; CBD, common bile duct; CT, computed tomography; DP, duodenal papilla; GB, gallbladder; HA, hepatic artery, Mid, mid-portion; PH, porta hepatis; PV, portal vein.

CBD diameter was measured at three different sites: at the porta hepatis (PH) level, at the duodenal papilla (DP) level, and mid-portion (Mid) between these points (Figure 1A). At the PH level, the hepatic artery, portal vein, and CBD were located in parallel, and the CBD diameter at the DP level was measured at the location where the CBD inserted into the duodenum. All measurements were performed with only axial plane using electronic caliper. The CBD diameter was measured from the leading edge to the trailing edge, including the ductal wall, and the widest diameter was measured perpendicular to its long axis (Figures 1B–D). CBD:Ao ratio was evaluated at PH, Mid, and DP levels. Ao diameter was measured on the axial plane and at the location where each CBD diameter was measured: PH, Mid, and DP level. For the Ao measurement, minor axis diameter of the vessel was measured (Figure 1E).

For intraobserver, reliability analysis, the CBD diameters at each level were measured twice by observer A. For interobserver reliability analysis, the CBD diameters at each level were measured by three different clinicians, observer A-C (Residents in the Veterinary Medical Imaging Department of the Teaching of Jeonbuk National University).

### 2.3. Statistics

All values were presented as the mean and standard deviation (SD). Since this study only included dogs without hepatobiliary disease, we established the upper limit of the normal CBD diameter instead of normal cutoff value. The mean + 1.96 SD value was used for the upper limit of the 95% reference range of the CBD diameter. One-way analysis of variance (ANOVA) was used to analyse the differences in CBD diameter, CBD: Ao ratio between BW groups and to analyse difference of CBD diameters between three different sites. Games-Howell and Scheffe's tests were used for *post ho*c analysis when the ANOVAs were significant (*p* < 0.05^*^). Simple linear regression analysis was applied to analyse the correlation between “BW and CBD diameter” and “age and CBD diameter.” The *t-*test was used to compare whether there was a difference of CBD diameter between sexes. Intraobserver and interobserver reliability for CBD diameter was assessed using the absolute agreement-type intraclass correlation coefficient (ICC) with a 95% CI. IBM Statistical Package for Social Sciences Statistics (SPSS; version 27.0, IBB Corp., Armonk, NY, USA) was used for all statistical analyses, and all experimental values were considered significant at *p* < 0.05^*^ or *p* < 0.001^**^.

## 3. Results

### 3.1. Animal

The dogs were of 32 different breeds, including Maltese (*n* = 59), mixed (*n* = 39), Poodle (*n* = 37), Yorkshire terrier (*n* = 16), Shih tzu (*n* = 16), Pomeranian (*n* = 15), Schnauzer (*n* = 13), Retriever (*n* = 11), Chihuahua (*n* = 9), Spitz (*n* = 9), Dachshund (*n* = 8), Beagle (*n* = 8), Cocker spaniel (*n* = 7), Bichon frise (*n* = 6), Pekingese (*n* = 5), French bulldog (*n* = 3), Samoyed (*n* = 3), Miniature pinscher (*n* = 2), Italian greyhound (*n* = 2), Shetland sheepdog (*n* = 2), Chow chow (*n* = 2), Cavalier King Charles spaniel (*n* = 1), Bull terrier (*n* = 1), Great Pyrenees (*n* = 1), Husky (*n* = 1), Laika (*n* = 1), Scottish terrier (*n* = 1), Pug (*n* = 1), Welsh corgi (*n* = 1), Shiba inu (*n* = 1), Australian shepherd (*n* = 1), and Boston terrier (*n* = 1); Maltese dogs were the most common. The mean BW was 7.85 kg (range: 1.55–29.5 kg). The average age of all dogs was 8.8 years (range: 0.5–17 years). The study included 144 male and 139 female dogs. The number of dogs in each BW group was as follows: Class 1, *n* = 119; Class 2, *n* = 99; Class 3, *n* = 35; and Class 4, *n* = 30. The body condition scores (BCS) of the included dogs were 4 or 5/9.

### 3.2. Reference range and differences in the CBD diameters for different BW groups

**Figure 2:**
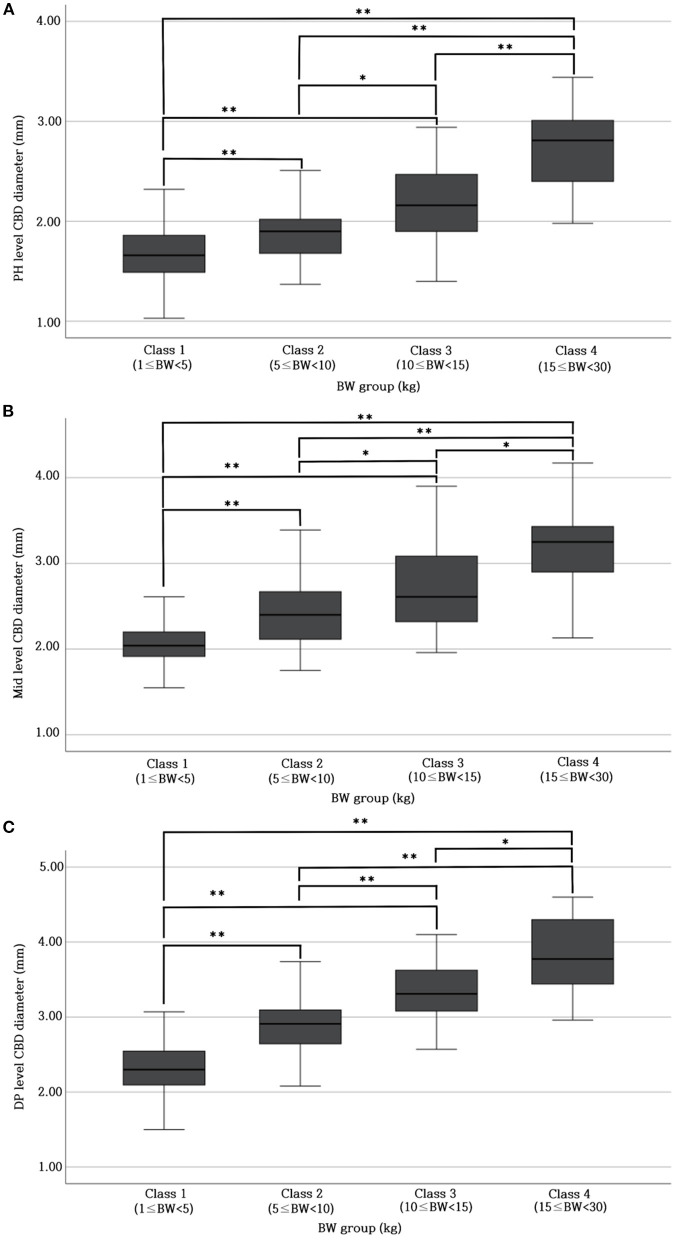
Differences in CBD diameter at PH level **(A)**, Mid-level **(B)**, and DP level **(C)** between different BW groups. There were significant differences (*p* < 0.05* or *p* < 0.001**) in CBD diameter at each level (PH, Mid, and DP level) among all BW groups. BW, body weight; CBD, common bile duct; DP, duodenal papilla; Mid, mid-portion; PH, porta hepatis.

Table 1 provides the normal reference range and upper limit of the CBD diameters at each level for each BW group. The CBD diameter at each level differed significantly (*p* < 0.05^*^ or *p* < 0.001^**^) among all BW groups (Figures 2A–C).

**Table 1:** Values of normal reference range of CBD at PH, Mid, and DP level for different BW groups.

**BW (kg) group**	**Number**	**CBD diameter Mean** ±**SD (mm)**			**95% reference intervals upper limit (mm)**		
		**PH**	**Mid**	**DP**	**PH**	**Mid**	**DP**
Class 1 (1 ≤ BW < 5)	119	1.69 ± 0.29	2.06 ± 0.25	2.33 ± 0.34	1.74	2.10	2.39
Class 2 (5 ≤ BW < 10)	99	1.92 ± 0.35	2.43 ± 0.37	2.90 ± 0.36	1.99	2.51	2.97
Class 3 (10 ≤ BW < 15)	35	2.20 ± 0.43	2.74 ± 0.52	3.35 ± 0.49	2.35	2.92	3.52
Class 4 (15 ≤ BW < 30)	30	2.79 ± 0.49	3.14 ± 0.44	3.83 ± 0.50	2.97	3.31	4.02

BW, body weight; CBD, common bile duct; DP, duodenal papilla; Mid, mid-portion; PH, porta hepatis; SD, standard deviation.

### 3.3. Correlations between the CBD diameters and BW

**Figure 3:**
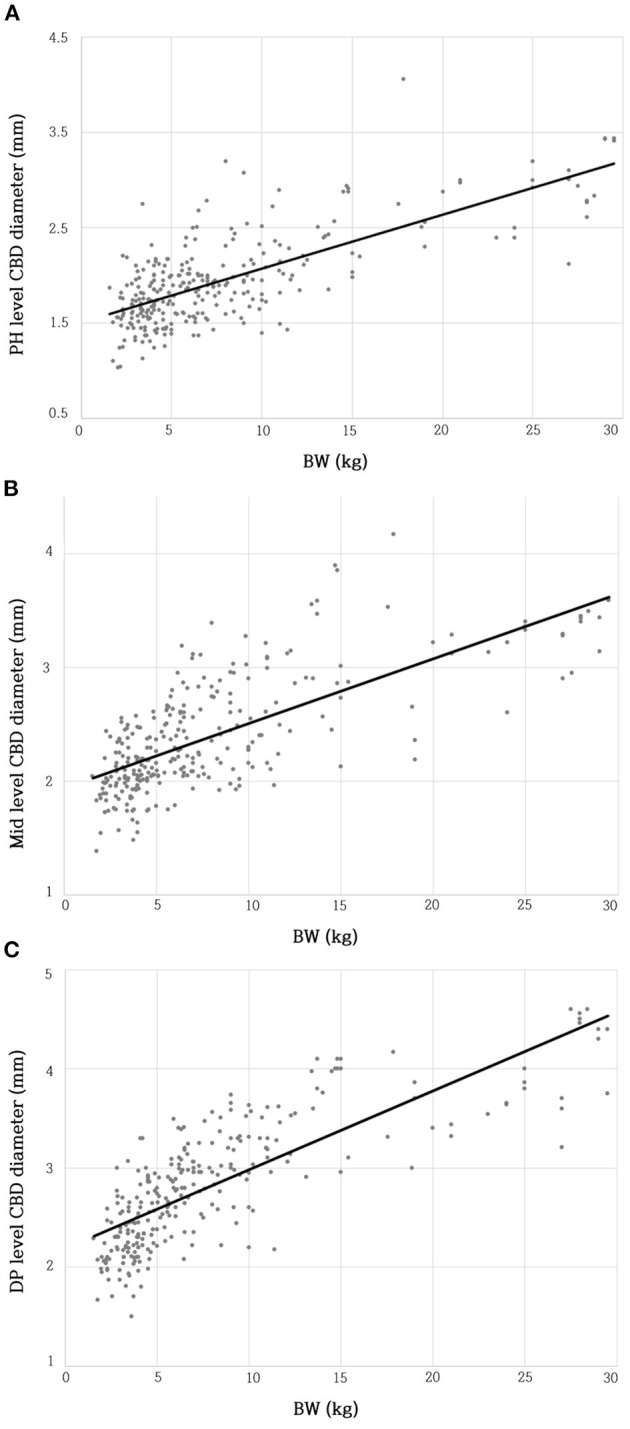
Relationship between BW and CBD diameter at PH level **(A)**, Mid-level **(B)**, and DP level **(C)**. There were moderate linear positive correlations between BW and CBD diameter at each level. BW, body weight; CBD, common bile duct; DP, duodenal papilla; Mid, mid-portion; PH, porta hepatis.

The BW and CBD diameter at the PH level showed a moderate positive linear correlation (R^2^ = 0.535; β = 0.057; *p* < 0.001^**^) (Figure 3A). The BW and CBD diameter at the Mid-level showed a moderate positive linear correlation (R^2^ = 0.511; β = 0.057; *p* < 0.001^**^) (Figure 3B). The BW and CBD diameter at the DP level showed a moderate positive linear correlation (R^2^ = 0.629; β = 0.079; *p* < 0.001^**^) (Figure 3C).

### 3.4. CBD: Ao ratio

**Figure 4:**
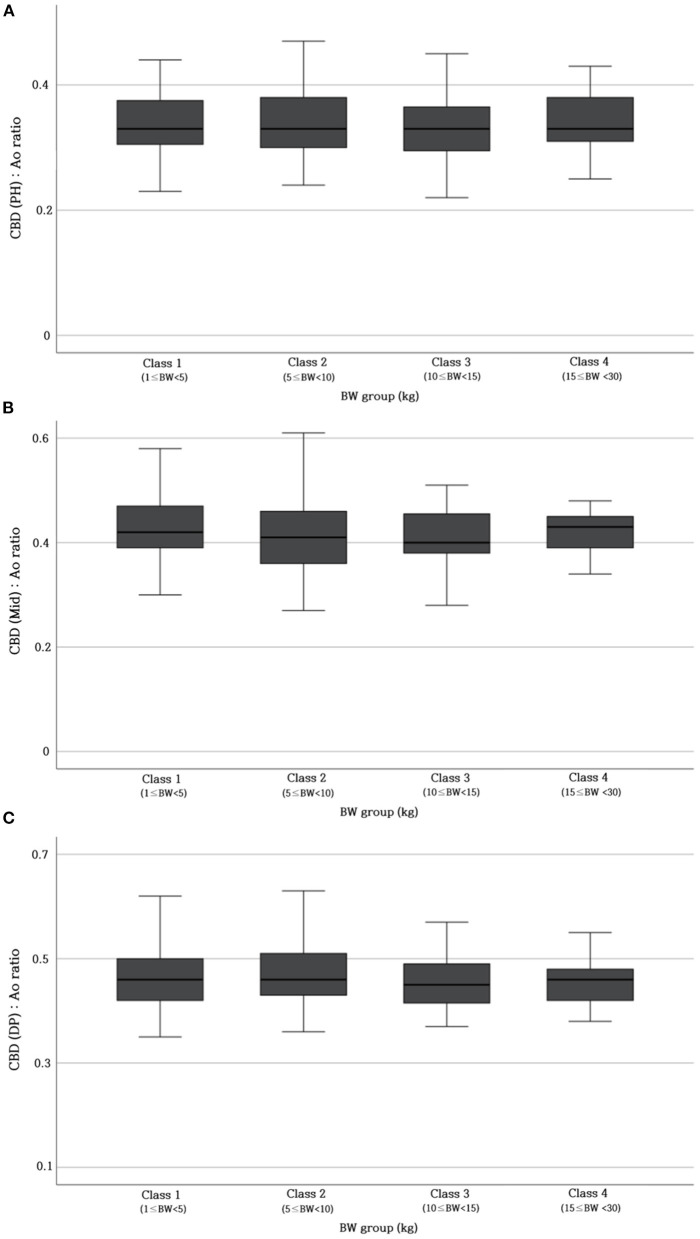
Differences in CBD: Ao ratio at PH level **(A)**, Mid-level **(B)**, and DP level **(C)** between different BW groups. No significant differences (*p* > 0.05) are found in the CBD: Ao ratio between the BW groups. Ao, abdominal aorta; BW, body weight; CBD, common bile duct; DP, duodenal papilla; Mid, mid-portion; PH, porta hepatis.

Table 2 provides the reference range of CBD: Ao ratio at three different sites. To verify whether this ratio was not affected by BW, the CBD: Ao ratio was compared between different BW groups. The ratio showed no significant difference at each site (p > 0.05) (Figure 4).

**Table 2:** Values of normal reference range of CBD: Ao ratio at PH, Mid, and DP level.

**Site**	**CBD: Ao ratio**
PH	0.34 ± 0.05
Mid	0.42 ± 0.06
DP	0.47 ± 0.06

Ao, abdominal aorta; CBD, common bile duct; DP, duodenal papilla; Mid, mid-portion; PH, porta hepatis.

### 3.5. Differences in the CBD diameters at the three different levels (PH, Mid, and DP level)

There was a significant difference in the mean CBD diameters between the three different levels (mean CBD diameter at PH; 1.95 ± 0.49 mm, Mid; 2.39 ± 0.50 mm, DP; 2.81 ± 0.63 mm; *p* < 0.001^**^).

### 3.6. Correlation between CBD diameter and age

Age showed no correlation with CBD diameter at the PH level (R^2^ = 0.000; β = −0.002; *p* = 0.841), Mid-level (R^2^ = 0.004; β = −0.008; *p* = 0.320), and DP level (R^2^ = 0.000; β = −0.003; *p* = 0.728).

### 3.7. Difference between the CBD diameters and sex

No significant differences in CBD diameters were observed in relation to sex at PH level (*t-*value = 0.762, *p* = 0.447), Mid-level (*t-*value = 0.613, *p* = 0.540) and DP level (*t-*value = 0.497, *p* = 0.620).

### 3.8. Intraobserver and interobsever reliability

Table 3 provides the first and the second measured CBD diameters at each level. The intraobserver reliability measured by ICC was 0.962 at the PH level, 0.997 at the Mid-level, and 0.981 at the DP level. The measured values of CBD diameter at each level showed almost perfect reliability for the two measurements. Table 3 also provides the CBD diameters measured by three clinicians (observer A-C) at each level. Interobserver reliability measured by ICC was 0.975 at the PH level, 0.958 at the Mid-level, and 0.956 at the DP level which were consistent with almost perfect reliability.

**Table 3:** Intraobserver and interobserver reliability for the CBD diameter at PH and DP level using ICC and their 95% CI.

**Parameter**		**Mean ±SD (mm)**	**ICC**	**95% CI**	***p* value**
Intraobserver (observer A)	**PH level**		0.962	0.952–0.970	*p* < 0.001^**^
	First	1.93 ± 0.50			
	Second	1.92 ± 0.52			
	**Mid–level**		0.997	0.997–0.998	*p* < 0.001^**^
	First	2.38 ± 0.50			
	Second	2.39 ± 0.50			
	**DP level**		0.981	0.973–0.986	*p* < 0.001^**^
	First	2.73 ± 0.63			
	Second	2.78 ± 0.65			
Interobserver	**PH level**		0.975	0.951–0.985	*p* < 0.001^**^
	Observer A	1.95 ± 0.49			
	Observer B	1.98 ± 0.51			
	Observer C	2.09 ± 0.48			
	**Mid–level**		0.958	0.861–0.980	*p* < 0.001^**^
	Observer A	2.39 ± 0.50			
	Observer B	2.57 ± 0.49			
	Observer C	2.62 ± 0.51			
	**DP level**		0.956	0.822–0.981	*p* < 0.001^**^
	Observer A	2.81 ± 0.63			
	Observer B	2.79 ± 0.65			
	Observer C	3.10 ± 0.63			

CI, confidence interval; DP, duodenal papilla; Mid, mid-portion between PH and DP level; ICC, intraclass correlation coefficient (absolute agreement); SD, standard deviation; PH, porta hepatis. Experimental values were considered significant at p < 0.001^**^.

## 4. Discussion

This study focused on the normal reference range in different BW groups and correlation between CBD diameter and BW using CT. Based on our findings, there was a significant difference in the CBD diameter in different BW groups. Moreover, positive linear correlation between CBD diameter and BW was observed. Thus, we devised a new method using Ao diameter, the CBD: Ao ratio, which is not affected by BW. To the best of the author's knowledge, this new method has not been reported in veterinary medicine.

To date, in veterinary medicine, specific reference range of CBD diameter using CT has not been established for different BW groups in normal dogs. A recent previous study evaluating the CBD diameter using CT have been reported for small breed dogs weighing <15 kg in 50 normal dogs (11). However, that study was performed only in a narrow range of BW and the number of dogs used was small. In our study, the BW range was wide (1.55–29.5 kg), and the number of dogs used was large with 283. In the previous study, the mean CBD diameter was 1.85 ± 0.63 mm at the PH level and 2.70 ± 0.51 mm at the DP level (11). Mean CBD diameters of the previous study were similar to those of Class 1 (PH; 1.69 ± 0.29, DP; 2.33 ± 0.34) and Class 2 (PH; 1.92 ± 0.35, DP; 2.90 ± 0.36), and Class 3 (PH; 2.20 ± 0.43, DP; 3.35 ± 0.49) in our study, but mean CBD diameters of Class 4 (PH; 2.79 ± 0.49, DP; 3.83 ± 0.50) were larger than those of the previous study (11). It was inferred that the reason for these differences was that the BW of Class 4 (15 kg≤BW < 30 kg) was larger compared to the BW used in previous study (11). Therefore, in a wide range of BW, it may be difficult to use the values of the previous study. Furthermore, results of our study showed significant differences in the CBD diameters for different BW groups and its values were significant higher in Class 4 (PH; 2.79 ± 0.49, Mid; 3.14 ± 0.44, DP; 3.83 ± 0.50) than Class 1 (PH; 1.69 ± 0.29, Mid; 2.06 ± 0.25, DP; 2.33 ± 0.34). Thus, we assumed that CBD diameter was affected by BW in normal dogs, and that different normal reference range of CBD diameters should be used for different BW groups.

BW is one of the most representative physiological indicators in dogs and humans. Our study showed positive linear correlations between CBD diameter and BW, which was similar to report in previous human medicine (14). However, other human studies have suggested that there was no association between CBD diameter and BW (15–17). We suppose that the reason for these results may be the differences in the variability of physiques between dogs and humans. In a human study, the participants were usually adults and their height (153.42–173.38 cm) and weight (39.15–63.65 kg) showed relatively small differences in physique (17). However, dogs vary significantly in physique between breeds, such as the Maltese and Great Pyrenees (18), causing significant differences and correlations in CBD diameter between dogs with large differences in physique. Additionally, obesity is an indicator that affects the physique index, but since the BCS of the dogs used in our study was ideal (BCS 4 or 5), it was inferred that obesity did not affect the results of our study (19). Moreover, in a previous study in veterinary medicine, no correlation was observed between BW and the CBD diameter at the PH level, although BW showed a mild correlation with the CBD diameter at the DP level (11). We inferred that the reason for these results may be because of differences such as number of samples, range of BW, and breeds.

Since the CBD diameter significantly differs according to BW, we attempted to devise a new indicator that can evaluate CBD diameter regardless of the BW. The aortic diameter has been used in ratio studies as a reliable landmark (20, 21). Due to the tortuous course of the aorta, it may appear elliptical, leading to an overestimation of the actual diameter when using the major axis of the ellipse (22). Therefore, minor axis diameter of the aorta was measured in this study. We investigated whether the CBD: Ao ratio showed a constant value in different BW groups. The results confirmed that the CBD: Ao ratio remained constant between different BW groups (*p* > 0.05). Therefore, this new index could be used regardless of the BW.

In previous veterinary study, CBD diameters showed significant differences at PH and DP level (11). Therefore, in our study, CBD diameters were measured at different sites; PH, Mid and DP level. There were significant differences in the CBD diameters for the three different sites, which is similar to the results of a previous veterinary medicine study. As a result, it is important to use different reference ranges for PH, Mid, and DP levels.

In this study, age showed no correlation with CBD diameters at each level. This result was similar with reports in previous human medicine (23). However, other human medicine studies have reported significant positive correlations between age and CBD diameter (14–17, 24–26). Although the reason for this discrepancy is not clear, the CBD diameter can be considered to have increased with age because the longitudinal smooth muscle band and the intervening connective tissue fragments were accompanied by loss of the reticulo-endothelial network of the ductal wall (16, 27). The reason for difference with that in humans was possibly because that the age range of humans was significantly wider (range: 18–85 years) than the relatively narrow age range in our study (range: 0.5–17 years) (17).

There was no significant difference in CBD diameter between sexes in our study. Several studies in human medicine emphasized that CBD diameters did not differ according to sex, which is same with our study (15–17).

While a previous veterinary study proposed 3 mm as the normal CBD diameter in dogs on US examination, our study showed that the normal CBD diameters in some dogs are larger than 3 mm (10). Several human studies have suggested that measurements of abdominal organ size were larger on CT compared to US (28, 29). The reason for such differences may be related to the characteristics of the imaging modality. While US can clearly distinguish specific abdominal organs from surrounding fat or other soft tissue, CT may have less clear organ contouring or delineation, which can lead to larger measurements on CT compared to US (28–30).

Intra- and interobserver reliability analyses were conducted to estimate reproducibility and reliability of measurement. We used ICC with 95% CI as described by Fleiss in which if the ICC value is 0.81–0.99, it is considered almost perfect agreement (31). In our study, intra- and interclass correlation coefficient showed almost perfect agreement. Therefore, through these results, it can be considered that the values of the reference ranges that we set are reliable.

Our study had several limitations. First, determination of the exact normal cutoff value for identifying abnormal conditions was not established because abnormal subjects were not included in this study. However, we established the upper limit of the normal CBD diameter for clinical recommendations. Future studies should aim to add and compare an abnormal group to set the normal cutoff value. Second, because of the retrospective nature of the study, CT machines used in this study were different. Some scan parameters of the images used in this study, including slice thickness, kVp, and mAs, were slightly different. However, since this study was conducted on a large scale with 283 dogs, it could be inferred that the difference due to the scan parameters would be relatively small. Lastly, the number of dogs in each BW group was unequal. Therefore, further studies should be conducted in a condition where the number of subjects in each BW group is similar.

In conclusion, based on our review of the literature, this is the first study to establish a normal reference range of the CBD diameter between different BW groups by using CT. Since the CBD diameter differs depending on BW, using a different normal reference range for different BW group is essential, while CBD: Ao ratio can be used valuable regardless of BW.

## Data availability statement

The original contributions presented in the study are included in the article/supplementary material, further inquiries can be directed to the corresponding author.

## Ethics statement

The animal study was reviewed and approved by Institutional Animal Care and Use Committee of Jeonbuk National University (Approval No. JBNU 2022-037). Written informed consent was obtained from the owners for the participation of their animals in this study.

## Author contributions

Conception and design, acquisition of data, and drafting the article: YK and HY. Analysis and interpretation of data, revising article for intellectual content, and final approval of the completed article: YK, SK, DK, DI, KL, and HY. Agreement to be accountable for all aspects of the work ensuring that questions related to the accuracy or integrity of any part of the work are appropriately investigated and resolved: YK, KL, and HY. All authors contributed to the article and approved the submitted version.
